# Plasma Circulating Tumor Epstein–Barr Virus for the Surveillance of Cancer Progression in Bone-Only Metastatic Nasopharyngeal Carcinoma

**DOI:** 10.3389/fonc.2022.860700

**Published:** 2022-06-10

**Authors:** Guo-Ying Liu, Wei-Xiong Xia, Zhuo-Fei Bi, Nian Lu, Wang-Zhong Li, Wei-Xin Bei, Hu Liang, Jun-Zhi Xie, Yi-Min Liu, He-Rui Yao, Yan-Qun Xiang

**Affiliations:** ^1^ Guangdong Provincial Key Laboratory of Malignant Tumor Epigenetics and Gene Regulation, Medical Research Center, Sun Yat‐sen Memorial Hospital, Sun Yat‐Sen University, Guangzhou, China; ^2^ Department of Nasopharyngeal Carcinoma, Sun Yat-sen University Cancer Center, Guangzhou, China; ^3^ Department of Oncology, Sun Yat‐sen Memorial Hospital, Sun Yat‐sen University, Guangzhou, China; ^4^ State Key Laboratory of Oncology in South China, Collaborative Innovation Center for Cancer Medicine, Guangdong Key Laboratory of Nasopharyngeal Carcinoma Diagnosis and Therapy, Sun Yat-sen University Cancer Center, Guangzhou, China; ^5^ Richard Montgomery High School at Rockville of Maryland, Rockville, MD, United States

**Keywords:** nasopharyngeal carcinoma, EBV-DNA, bone-only metastasis, surveillance, cancer progression

## Abstract

**Background:**

Plasma Epstein–Barr virus DNA (EBV-DNA) is a sensitive and specific biomarker for nasopharyngeal carcinoma (NPC). We investigated whether longitudinal monitoring of EBV-DNA could accurately detect clinical disease progression in NPC patients with bone-only metastases.

**Methods:**

In this retrospective study, a total of 105 patients with bone-only metastatic NPC who were treated with platinum-based first-line chemotherapy were enrolled. Undetectable EBV-DNA after first-line chemotherapy was defined as a biochemical complete response (BCR). The correlation of the EBV-DNA dynamic status with overall survival (OS) and progression-free survival (PFS) was determined by Cox regression. The correlation between non-normalized EBV-DNA period and PFS period was determined.

**Results:**

After a median follow-up time of 53.4 months [Interquartile range (IQR): 42.8–80.6], 64 patients had disease progression. Thirty-nine of 105 patients (37.1%) had a BCR at all follow-up time points, and none of these 39 patients had disease progression, corresponding to a negative predictive value (NPV) of 100%. Sixty-six patients had a detectable EBV-DNA during surveillance, with 64 diagnosed as disease progression at the last follow-up, for a positive predictive value (PPV) of 97.0%. Actuarial 3-year OS rates were 45.0% for patients with detectable EBV-DNA during posttreatment surveillance and 100% for patients with undetectable EBV-DNA. Lastly, median lead time between non-normalized EBV-DNA and clinically proven progression was 5.87 ± 0.67 months.

**Conclusions:**

Taken together, EBV-DNA provided predictive value for the bone-only metastatic NPC patients. The results should be validated in prospective randomized studies.

## Introduction

Nasopharyngeal carcinoma (NPC) is characterized by a distinct geographical distribution and mainly prevalent in Southeast Asia, especially in Southern China (1–3). Bone is one of the most common sites in patients with metastatic NPC and is an important cause of morbidity and disability (4). How to best monitor the response of bone metastases from NPC to systemic therapy is a critical issue in clinical practice. To effectively manage patients with bone metastases, it is essential to develop consistent, reproducible, and reliable methods.

Recently, to detect metastatic bone lesions, the mainstay of the evaluation method remains bone scintigraphy. In some bone metastasis cases, x-ray, magnetic resonance imaging (MRI), computed tomography (CT), and positron emission tomography (PET) are used to improve the diagnostic accuracy (5, 6). Nevertheless, despite continuous improvements in imaging technology, bone metastases remain inadequate, as increased sclerosis detected on specific imaging or bone scan flashes associated with treatment response may be mistaken for progress (7, 8). Yet, a consensus has not been reached regarding monitoring during and post therapy for bone-only metastases in this clinical setting. 

Blood-based surveillance tests have the potential to facilitate early detection of cancer recurrence, as recently demonstrated by personalized circulating tumor DNA testing for bladder, breast, and colorectal cancers (9–11). Plasma Epstein–Barr virus (EBV) infection is detectable in approximately 90% of patients at diagnosis (12, 13); therefore, circulating tumor EBV-DNA has become an established biomarker for NPC (14). Dynamic changes in EBV-DNA levels are correlated with treatment response in patients with localized or metastatic NPC (15). The clinical utility of longitudinal EBV-DNA monitoring for monitoring of disease progression after first-line chemotherapy has not been established in bone-only metastatic NPC.

This study aimed to determine the value of longitudinal EBV-DNA as a biomarker in monitoring disease progression in patients with bone-only metastatic NPC who received first-line chemotherapy.

## Materials and Methods

### Patients

This is a retrospective cohort study based on patient information recorded in the institutional database. We evaluated data from patients with bone-only *de novo* metastatic NPC who received first-line platinum-based chemotherapy at Sun Yat-sen University Cancer Center (SYSUCC) between March 2010 and December 2018. Patients who fulfilled the following inclusion criteria were enrolled: 1) *de novo* metastatic NPC with bone-only metastatic disease; 2) receiving platinum-based chemotherapy as first-line treatment at least for two cycles; 3) all patients had histologically proven NPC without prior treatment for metastasis; 4) baseline EBV-DNA >0 copies/ml. Patients with a previous history of carcinoma during the past 5 years were excluded. This study was approved by the Research Ethics Committee of SYSUCC. A written consent that individual medical data might be utilized for future medical studies was routinely obtained as a standard procedure for patients treated in this center.

### Investigation

Peripheral blood was collected in an Ethylenediaminetetraacetic acid (EDTA) tube from patients, and the samples were centrifuged at 1,600× g for 15 min for isolation of plasma. Plasma EBV-DNA concentrations were measured by a qPCR assay. The detailed methodology for detecting plasma EBV-DNA is described in **Supplementary Material 1**. Peripheral blood for baseline estimation of EBV-DNA level was collected before the administration of chemotherapy. Blood specimens were collected pretreatment, every cycle during chemotherapy, approximately every 3 months for years 1–2, and then every 6 months for years 3–5.

### Treatment

All patients received platinum-based chemotherapy as first-line treatment. Definitive radiotherapy targeting both the primary tumor and its regional lymph nodes was administered to some patients. Local treatment of metastatic lesions to control local symptoms and eliminate metastases in the bone was allowed. The local treatment of metastatic primary tumor and its regional lymph node lesions was based on physician’s discretion, given a deemed clinical benefit. Detailed information on treatment is available in **Supplementary Material 1**.

### Outcome and Follow-Up

The negative predictive value (NPV) and positive predictive value (PPV) for EBV-DNA monitor to identify patients with disease progression of patients with bone-only metastasis were the primary endpoint. The secondary endpoint was overall survival (OS) and progression-free survival (PFS). OS, is defined as the time from the diagnosis of distant metastasis to the date of death or of last follow-up. PFS, is defined as the time from the day of diagnosis to evident tumor progression on any radiographic examination or clinical progression or death by any causes. Biochemical complete response (BCR) is defined as an EBV-DNA=0 copies/ml (undetectable) after the first-line chemotherapy. The date of the last follow-up was defined as the latest image study and/or clinic visiting and/or telephone follow-up. Patients were followed up every 2 months during systemic chemotherapy and every 3 months after completion of chemotherapy until disease progression or death. Duration is calculated from the beginning of treatment to each event or last follow-up.

### Statistical Analysis

Baseline characteristics were compared between different EBV-DNA surveillance statuses using Kruskal–Wallis and χ^2^ tests. Actuarial survival rates of significant risk factors were estimated using the Kaplan–Meier method, and survival curves were compared by the log-rank test. For multivariable analysis, a forward stepwise Cox proportional hazards model was used, with P < 0.05 determining which variables should be entered into the model at each step. The nonparametric Spearman rank correlation coefficient (rs) was used to measure the correlation between the undetectable EBV-DNA period and the survival period. Linear regression analysis was performed through the origin of the plot evaluating the PFS period as a function of undetectable EBV-DNA period. Statistical analysis was performed using version 22.0 of the Statistical Package. All tests were two-sided; P < 0.05 was considered significant.

## Results

### Patient Cohorts

**Figure 1:**
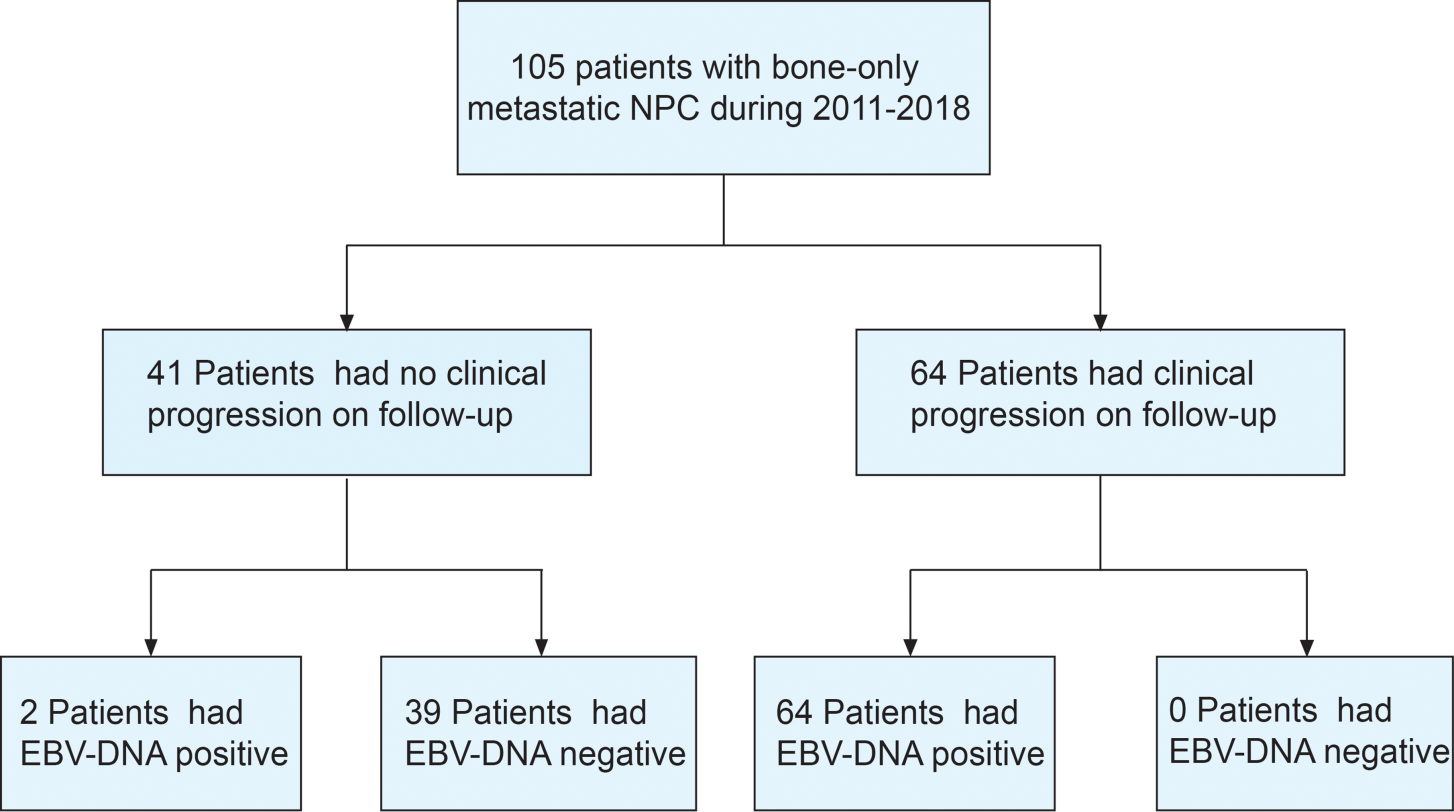
Flowchart of the different groups according to EBV-DNA kinetics.

Between March 2011 and December 2018, 105 bone-only metastatic NPC patients who received first-line platinum-based chemotherapy were enrolled (**Figure 1**). The median age was 45 years, most of the patients were men (88.6%), and with ECOG PS of 0–1 (91.4%) and WHO type III histology (99.0%). Most patients had multiple sites of metastases (69.5%). The clinical characteristics of the study population are shown in **Table 1**.

**Table 1:** Patient characteristics.

Characteristic	No. of patients	Undetectable surveillance (n = 39)	Detectable surveillance (n = 66)	P value
Gender				0.758
Men	93 (88.6%)	34 (87.2%)	59 (89.4%)	
Women	12 (11.4%)	5 (12.8%)	7 (10.6%)	
Age				0.312
≤45 years	57 (54.7%)	24 (61.5%)	33 (50.0%)	
>45 years	48 (45.3%)	15 (38.5%)	33 (50.0%)	
ECOG PS				0.479
≥90	96 (91.4%)	37 (94.9%)	59 (89.4%)	
≤80	9 (8.6%)	2 (5.1%)	7 (10.6%)	
Histology				1.000
II	1 (1.0%)	0	1 (1.5%)	
III	104 (99.0%)	39 (100%)	65 (98.5%)	
T stage				0.758
T1–2	12 (11.4%)	5 (12.8%)	7 (10.6%)	
T3–4	93 (88.6%)	34 (87.2%)	59 (89.4%)	
N stage				0.563
N0–1	14 (13.3%)	4 (10.3%)	10 (15.4%)	
N2–3	91 (86.7%)	35 (89.7%)	56 (84.8%)	
Smoking status				1.000
Yes	31 (29.5%)	11 (28.2%)	20 (30.3%)	
No	74 (70.5%)	28 (71.8%)	46 (69.7%)	
Number of metastases				0.030
Single	32 (30.5%)	17 (43.6%)	15 (22.7%)	
Multiple	73 (69.5%)	22 (56.4%)	51 (77.3%)	
First-line chemotherapy				0.261
PF	32 (30.5%)	12 (30.8%)	20 (30.3%)	
TP	31 (29.5%)	9 (23.1%)	22 (33.3%)	
TPF	39 (37.1%)	18 (46.1%)	21 (31.8%)	
GP	3 (2.9%)	0	3 (4.6%)	
Local consolidative radiotherapy				0.057
Bone and locoregional	11 (10.5%)	2 (5.1%)	9 (13.6%)	
Locoregional	23 (21.9%)	13 (33.3%)	10 (15.2%)	
None	71 (67.6%)	24 (61.5%)	47 (71.2%)	
Baseline EBV-DNA				0.157
≤14,900 copies/ml	54 (51.4%)	24 (61.5%)	30 (45.5%)	
>14,900 copies/ml	51 (48.6%)	15 (38.5%)	36 (54.5%)	

ECOG PS, Eastern Cooperative Oncology Group performance status; PF, cisplatin and 5-fluorouracil; TP, taxane and cisplatin; TPF, taxane, cisplatin, and 5-fluorouracil; GP, gemcitabine and cisplatin.

At the cutoff date of June 28, 2021, the median follow-up was 53.4 months (IQR: 42.8–80.6). Sixty-four patients had disease progression; 18, new bone lesion; 12, local recurrence (nasopharyngeal/cervical lymph nodes); 2, death; 32, distant metastasis other than bones. At the time of the analysis, 42 of the 105 patients (40.0%) died of disease. The 3-year OS rate for the entire patient cohort was 66.7%, and the 3-year PFS rate was 39.9%.

### EBV-DNA After First-Line Chemotherapy and Survival

**Figure 2:**
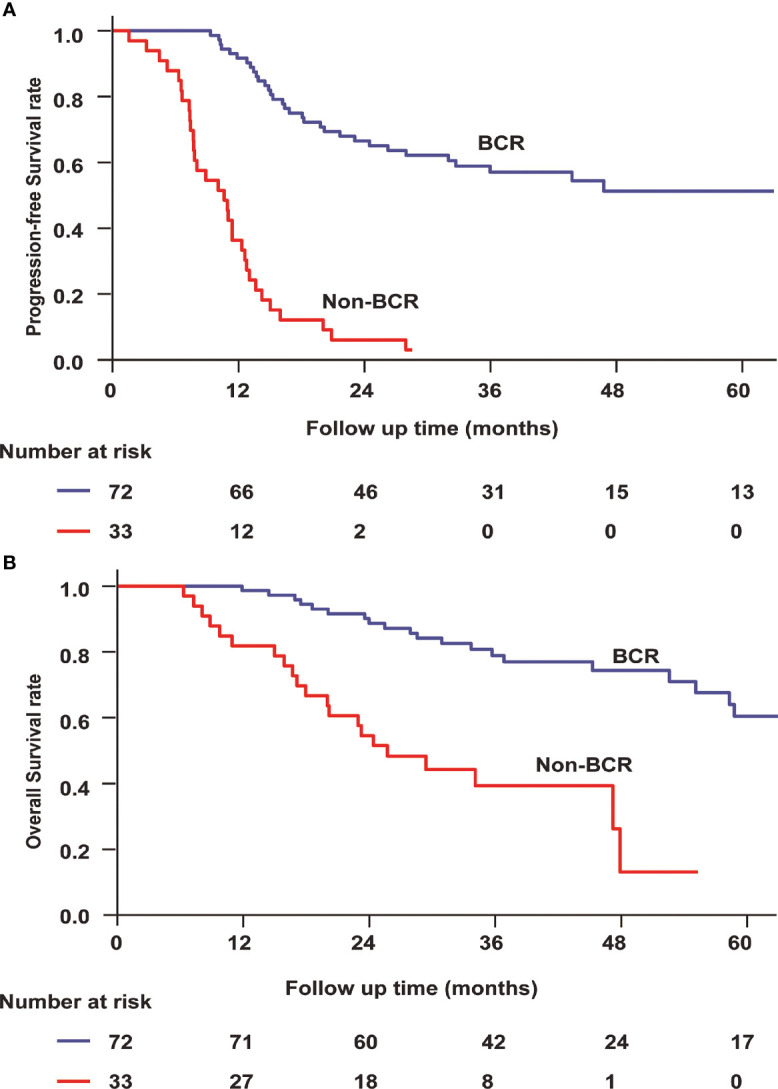
Progression-free survival **(A)** and overall survival **(B)** for bone-only metastatic nasopharyngeal carcinoma according to EBV-DNA kinetics. BCR, biochemical complete response.

After first-line chemotherapy, 72 patients (70.9%) reached a BCR, while the remaining 33 patients were defined as non-responders. We found a significant difference between EBV-DNA level after chemotherapy and clinical relapse. Median PFS was not reached in the cohort of BCR patients but only 10.6 months (95% CI 7.29–14.0) for non-responders (P < 0.001, **Figure 2A**). Median OS was not reached among BCR patients but only 25.7 months (95% CI 17.4–34.1, P < 0.001) in non-responders (**Figure 2B**).

According to the different treatments, we divided the patients into two subgroups. One group included patients who received locoregional radiotherapy, while another group included patients who did not receive locoregional radiotherapy. Among patients who received locoregional radiotherapy, the BCR (log rank P = 0.046) was a prognostic factor for OS. Similarly, the BCR (log rank P < 0.001) remained a predictor of improved OS among patients who did not receive locoregional radiotherapy.

### Longitudinal EBV-DNA Monitoring

**Figure 3:**
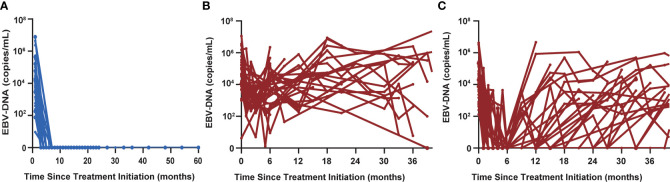
**(A)** Thirty-nine of 105 patients had undetectable EBV-DNA levels at all posttreatment surveillance time points. **(B)** Thirty-three non-responders still had detectable EBV-DNA signal during posttreatment surveillance. **(C)** Thirty-three patients who initially had a biochemical complete response had a detectable EBV-DNA test during posttreatment surveillance.

Thirty-nine of 105 patients (37.1%) had undetectable EBV-DNA at all follow-up time points (**Figure 3A**). None of these 39 patients was diagnosed with progressive disease, corresponding to an NPV of 100%. Thirty-three non-responders still had detectable EBV-DNA signal during posttreatment surveillance (**Figure 3B**). In addition, 33 patients who initially had a BCR had a detectable EBV-DNA test during posttreatment surveillance (**Figure 3C**). Median time to detectable EBV-DNA signal was 8.8 months after first-line chemotherapy. Median EBV-DNA level at the time of the first abnormal blood test was 7,475 copies/ml plasma (range, 45–5,000,000 copies/ml). Sixty-four of these 66 patients have been diagnosed with disease progression, for a PPV of 97.0%.

**Figure 4:**
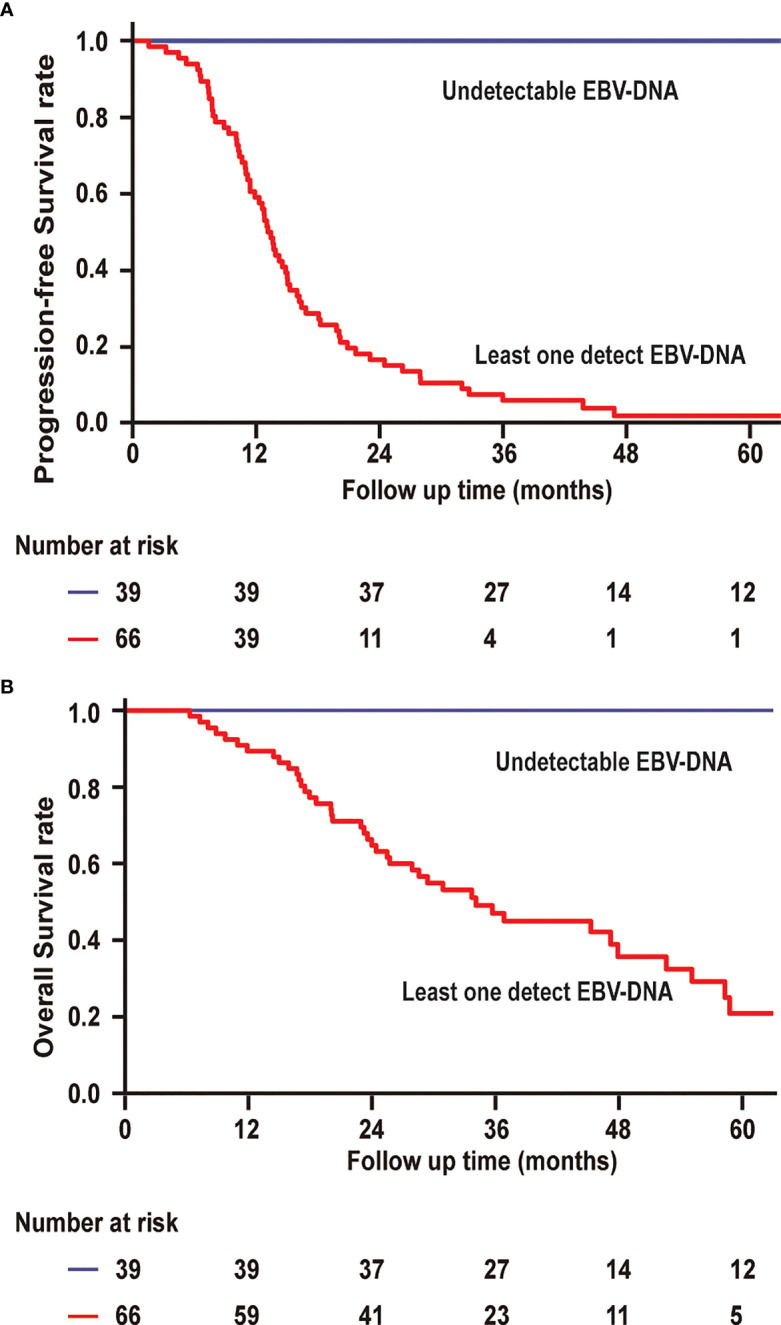
**(A)** Progression-free survival and overall survival **(B)** of patients with undetectable EBV-DNA at all surveillance time points vs. patients with at least one EBV-DNA blood test.

The 3-year PFS and 3-year OS rates were 6.1% and 45.0% for patients with a detectable EBV-DNA blood test during posttreatment surveillance, respectively, whereas these were 100% and 100% for the remaining patients with undetectable EBV-DNA (P < 0.001; **Figures 4A, B**), respectively. Univariate analysis of PFS among patients with bone-only metastatic NPC revealed that the number of metastasis [hazard ratio (HR), 1.950 (95% CI, 1.076–3.533); P = 0.028], baseline EBV-DNA [HR, 1.736 (95% CI, 1.058–2.849); P = 0.029], and EBV-DNA dynamic status [HR, 120.308 (95% CI, 15.036–962.644); P < 0.001] were significant predictors. Multivariate analysis based on the results of univariate analysis demonstrated that only the EBV-DNA dynamic status remained independent significant prognostic factors for PFS. Univariate analysis of OS revealed that baseline EBV-DNA [HR, 1.904 (95% CI, 1.020–3.551); P = 0.043] and EBV-DNA dynamic status [HR, 63.543 (95% CI, 5.194–777.361); P = 0.001] were significant predictors. Only the EBV-DNA dynamic status remained an independent significant prognostic factor for OS in multivariate analysis.

We analyzed EBV-DNA kinetics in a subset of 66 patients in whom a follow-up blood test was available after an initially positive EBV-DNA test during posttreatment surveillance. Among the 64 patients who developed disease progression, EBV-DNA levels remained elevated at a subsequent time point in all patients. In contrast, 2 patients who did not develop disease progression had clearance of EBV-DNA by the subsequent blood collection time points without treatment. These results indicate that majority of patients who have persistently elevated circulating EBV-DNA are associated with recurrent or metastatic disease, whereas approximately 3.0% of the patients in our cohort had a transient EBV-DNA level elevation during posttreatment surveillance that spontaneously resolved without intervening treatment and any clinical evidence of disease progression.

### Early Detection of Recurrence With EBV-DNA

**Figure 5:**
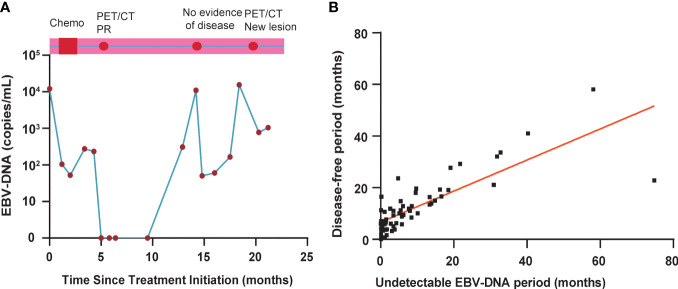
**(A)** EBV-DNA kinetics and clinical surveillance imaging results for a study patient with a 9-month interval from the first detectable EBV-DNA blood test result and clinical diagnosis of disease progression. PET, positron emission tomography; CT, computed tomography; PR, partial response. **(B)** Correlation between normalized EBV-DNA period and progression-free survival period in 64 patients with disease progression.

Among 46 of these 64 disease progression patients, abnormal EBV-DNA was detected before disease progression diagnosis during routine clinical follow-up. In 16 of these 64 patients, abnormal EBV-DNA was detected while the diseases were not noted to progress at the same time. Two patients had undetectable EBV-DNA before disease progression, but it was detectable at approximately 12.3 months after recurrence diagnosis. One patient demonstrated a 9-month lead time by EBV-DNA (**Figure 5A**). This patient achieved a radiographic partial response by MR scans of the head and neck and CT scans of chest and abdomen after completing chemotherapy, with a corresponding clearance of EBV-DNA levels in plasma. The patient developed EBV-DNA recurrence 3 months later, which was persistently elevated for three additional blood sampling time points afterward. During this period, the patient underwent PET/CT scans or MR scans of the head and neck and CT scans of the chest and abdomen that did not identify disease progression until the third abnormal EBV-DNA test result with new bone metastases after undergoing a PET/CT.

To assess the effect of undetectable EBV-DNA on PFS in bone-only metastatic NPC, we further analyzed the correlation between undetectable EBV-DNA period and PFS period in 64 patients who experimented disease progression. As shown in **Figure 5B**, the correlation between undetectable EBV period and PFS period is significant with the rs value of 0.76 (P < 0.001). The undetectable EBV-DNA period and PFS period was used to determine by a linear regression at the origin. The estimated intercept of the conversion factor for undetectable EBV-DNA period and PFS period was 5.87 ± 0.67 months.

## Discussion

The current study showed that circulating EBV-DNA is a sensitive marker of tumor burden in patients with bone-only metastatic NPC. Serial circulating EBV-DNA surveillance during follow-up could detect disease progression approximately 6 months prior to conventional radiography of bone-only metastatic NPC. To our knowledge, this is the first study to demonstrate the role of EBV-DNA in predicting disease progression of bone-only metastatic NPC.

Bone scintigraphy is considered the gold standard for monitoring metastatic bone lesions (6, 16, 17). However, bone scanning to identify bone metastases can confirm the remission of disease but is limited by low specificity and not an optimal way to assess a therapeutic response in bone lesions. PET imaging can improve specificity, but its sensitivity is low and the false negative rate is unacceptable (18, 19). Both imaging modalities are further restricted by cost, radiation exposure, invasiveness, and limits on the frequency of use. A blood-based surveillance test can overcome these limitations.

Circulating EBV-DNA has emerged as a biomarker of disease recurrence in patients with locally invasive or metastatic NPC (2). We have demonstrated that bone-only metastatic NPC patients with positive EBV-DNA after first-line chemotherapy had poor outcomes, with an estimated 3-year PFS of 47% vs. 76% of patients with a negative EBV-DNA after chemotherapy (HR, 3.8; P < 0.001). These results are consistent with previous studies in patients with metastatic and locally advanced NPC. In the present study, we also demonstrated the low probability of disease progression in bone-only metastatic NPC patients with persistently negative EBV-DNA throughout follow-up (0/39 patients; 0%). Our findings suggest that circulating EBV-DNA monitoring achieves better sensitivity for disease progress detection in the posttreatment setting for bone-only metastatic NPC patients. Our findings suggest that circulating EBV-DNA could be used as a possibility for an exclusion test to identify patients with fewer radiological examinations or follow-up. This finding could also substantially minimize radiation exposure and the costs associated with radiological tests during post-chemotherapy surveillance.

In terms of specificity, circulating EBV-DNA was positive in 2 of 66 (3%) patients who did not experience disease progression throughout follow-up. In repeated follow-up reanalysis, EBV-DNA was not detectable in either of them. We cannot rule out that these false positives are the result of some unrecognized technical artifacts and different testing methods. Positive EBV-DNA samples highlight the need to confirm low levels of positivity. Another possibility is that these patients may have experienced a subclinical relapse of immunological clearance. Additional studies are needed on the dynamics of EBV-DNA and its correlation with immune-related biomarkers for this subset of patients.

Unlike radiographic imaging interpretations, blood samples measurements are readily available during routine follow-up. Clearly, circulating EBV-DNA monitoring will be an important part of following patients with bone-only metastases to monitor progression. This leading time may allow for earlier implementation of other palliative strategies. Ideally, such a personalized surveillance strategy for each patient would allow for earlier detection of progression while minimizing radiological detection. Whether the leading time is associated with a better clinical outcome with earlier implementation of other palliative strategies is needs to be validated by larger prospective studies.

Locoregional radiation over chemotherapy is known to significantly improve OS in metastatic NPC (20), especially in oligometastatic disease. In this study, patients with EBV-DNA clearance were more likely to receive locoregional treatment. Furthermore, we performed a subgroup analysis to investigate whether the EBV-DNA clearance affects survival under different treatments. Meanwhile, our findings suggested that metastatic NPC patients with EBV-DNA clearance have a higher survival rate regardless of local radiotherapy.

Some limitations of this study should be mentioned. The study was limited by the sample size. Among others, the analysis was affected by the limitations and biases inherent in observational retrospective studies. In addition, this study is a single center study with a limited number of patients, so it is impossible to conduct a reliable multivariate analysis of the data. Prospective studies of EBV-DNA are still needed to determine whether or not the leading time in our study is insufficient used as a guide other curative or palliative strategies. Even for the same analysis using the same procedure but not coordinated, the differences between laboratories are relatively large. Therefore, it is necessary to standardize the detection of this biomarker in prospective studies.

## Conclusion

Taken together, circulating EBV-DNA provided valuable information regarding the progression of bone-only metastatic NPC patients. The clinical impact should be confirmed in prospective studies.

## Data Availability Statement

Our raw database has been validated by uploading the key raw data onto the Research Data Deposit (RDD) public platform (www.researchdata.org.cn), with the approval RDD number RDDA2022935660.

## Ethics Statement

The study was approved by the Institutional Review Board of Sun Yat-Sen University Cancer Center. Individual written informed consent was obtained from all participating patients.

## Author Contributions

Y-QX, H-RY and Y-ML designed the study. G-YL developed the methodology of study. G-YL, Z-FB, W-XB, W-ZL, NL, HL, J-ZX and W-XX participated in the acquisition of data. G-YL and W-ZL analyzed and interpreted the data. G-YL wrote the manuscript. All authors reviewed and revised the manuscript.

## Funding

This study was mainly supported by the National Natural Science Foundation of China (Nos. 82173232, 81802712, and 82002855).

## Conflict of Interest

The authors declare that the research was conducted in the absence of any commercial or financial relationships that could be construed as a potential conflict of interest.

## Publisher’s Note

All claims expressed in this article are solely those of the authors and do not necessarily represent those of their affiliated organizations, or those of the publisher, the editors and the reviewers. Any product that may be evaluated in this article, or claim that may be made by its manufacturer, is not guaranteed or endorsed by the publisher.
