# Supplementary material for: Plasma Circulating Tumor Epstein–Barr Virus for the Surveillance of Cancer Progression in Bone-Only Metastatic Nasopharyngeal Carcinoma
Source: Front Oncol. 2022 Jun 10;12:860700. doi: 10.3389/fonc.2022.860700 (PMC9226300; doi:10.3389/fonc.2022.860700)
Supplement: Supplementary file 1 [file DataSheet_1.docx]

**The supplementary data**

**The methodology of detecting plasma EBV DNA:**

Peripheral blood samples (3 ml) from all NPC patients were collected in EDTA tubes and centrifuged at 1600x g for 15 min to separate plasma and PBC. Plasma DNA was extracted using the QIAamp Blood Kit (Qiagen, Hilden, Germany) and stored at –80°C until further processing. A total of 500 μl of plasma sample per column was used for DNA extraction, and DNA was eluted from the extraction column using a final elution volume of 50 μl.

A real-time quantitative PCR system was developed to detect plasma EBV DNA in the BamHI-W region of the EBV genome. The sequence was determined by amplification primers W-44F (5'-AGT CTC TGC CTC AGG GCA-3') and W-119R (5'-ACA GAG GGC CTG TCC ACCG-3') and dual-labeled fluorescent probe W-67T (5'-[FAM]CAC TGT CTG TAA AGT CCA GCC TCC[TAMRA]-3').

**Treatment:**

All eligible patients received one of the following platinum-based chemotherapy regimens as the first-line treatment: (1) PF: cisplatin (80-100 mg/m^2^ intravenously on Days 1 of a 21-day cycle) plus 5-fluorouracil (500 mg/m^2^ continuous intravenously infusion on Days 1–5 of a 21-day cycle); (2) TP: paclitaxel (175-200 mg/m^2^ intravenously on Day 1 of a 21-day cycle) or docetaxel (70-75 mg/m^2^ intravenously on Day 1 of a 21-day cycle) plus cisplatin (75-80 mg/m^2^ intravenously on Days 1 of a 21-day cycle); (3) TPF: paclitaxel (135 mg/m^2^ intravenously on Day 1 of a 21-day cycle) or docetaxel (60 mg/m^2^ intravenously on Day 1 of a 21-day cycle) plus cisplatin (60 mg/m^2^ intravenously on Days 1 of a 21-day cycle) plus 5- fluorouracil (300 mg/m^2^, continuous intravenously infusion on Days 1–5 of a 21-day cycle); (4) GP: Gemcitabine (75-80 mg/m^2^ intravenously on Day 1 of a 21-day cycle) plus cisplatin (80-100 mg/m^2^ intravenously on Days 1 of a 21-day cycle).

After first-line chemotherapy, 34 patients and 11 patients received locoregional radiotherapy and metastasis radiotherapy, respectively. The accumulated radiation dose to the primary tumor was 66-70 Gy. The metastatic lymph node-positive received radiotherapy to a total dose of 60-66 Gy, and 45-60 Gy for the metastatic sites, respectively. Radiotherapy was given to all metastatic bones in the 11 patients, and all of them had <3 metastatic bones involved. The metastatic sites receiving radiotherapy were scapula, sacrum, ilium, cervical spine, lumbar spine and thoracic spine. All patients were given 5 daily fractions per week, at approximately 2 Gy/d.
